# R-Loop Formation *In Trans* at an AGGAG Repeat

**DOI:** 10.1155/2013/629218

**Published:** 2013-08-26

**Authors:** Kazuya Toriumi, Takuma Tsukahara, Ryo Hanai

**Affiliations:** ^1^Department of Life Science and Research Center for Life Science, Rikkyo (St. Paul's) University, 3-34-1 Nishi-Ikebukuro, Toshima-ku, Tokyo 171-8501, Japan; ^2^Tokyo Metropolitan Institute of Medical Science, 2-1-6 Kami-Kitazawa, Setagaya-ku, Tokyo 156-8506, Japan

## Abstract

Formation of RNA-DNA hybrid, or R-loop, was studied *in vitro* by transcribing an AGGAG repeat with T7 RNA polymerase. When ribonuclease T1 was present, R-loop formation *in cis* was diminished, indicating that the transcript was separated from the template and reassociated with it. The transcript was found to form an R-loop *in trans* with DNA comprising the AGGAG repeat, when the DNA was supercoiled. Results of chemical modification indicated that the duplex opened at the AGGAG repeat under negative supercoiling.

## 1. Introduction

The DNA-dependent RNA polymerase separates product RNA from its template DNA, thereby making the RNA available to ensuing cellular processes [1, 2]. The polymerase possesses structural elements for this separation and the RNA transcript is extruded through a hole of the polymerase molecule [3–6]. Despite this “separator” function of the polymerase, some transcripts have been known to anomalously form an RNA:DNA hybrid, or an R-loop, with their template, and R-loops have been implicated in a number of biological processes [7]. One classical example is the *colE1* replication origin, where the RNA in the R-loop serves as the primer of DNA replication [8]. A more recent example is the *FLOWERING LOCUS C* of *Arabidopsis thaliana*, where an R-loop at the promoter of the *COOLER* gene represses the transcription of the gene [9]. In addition to the “separator” function of the RNA polymerase, cellular processes ensuing transcription, such as splicing and RNA export, also serve to sequester RNA from the template DNA. Compromising such a function has been shown to result in hyperrecombination [7, 10].

R-loops have also been observed at G-rich repetitive sequences in immunoglobulin class-switch regions, although R-loop *per se* is not considered to trigger class-switching [11–16]. R-loop formation at a class-switch region was first shown for the murine S_*α*_ region [11]. Transcription of supercoiled plasmid DNA containing a 2.3 kb fragment of the region resulted in relaxation of the DNA, and the relaxation was reversed by RNase H treatment, which indicated R-loop formation. The relaxation was dependent on the direction of transcription. Radiolabeling experiments showed that the R-loop formed also when the template was linearized and that the RNA content in the R-loop was solely A and G [12]. The latter result, together with the length of the RNase A-resistant RNA bound to the DNA, indicated that the R-loop formed at the 28 tandem repeats of AGGAG in the S_*α*_ region.

Despite the simple repetitive nature of the S_*α*_ sequence, the exact mechanism of the R-loop formation has not been known. In this report, we present experimental results *in vitro* on an AGGAG repeat to address the following two questions. Firstly, it is not known whether the RNA is separated from and reassociate with the template or a gross structural change takes place in the polymerase molecule for the transcript to stay on the template. The latter possibility cannot be ruled out *a priori*, because the R-loop was found to form in the presence of ribonuclease (RNase) A *in vitro* [12]. Secondly, it is not known what structure in the DNA and/or the RNA leads to the R-loop formation: it is not known what allows the transcript to reassociate with its template or what forces the polymerase into an altered conformation in which the transcript can stay on the template.

## 2. Materials and Methods

### 2.1. Plasmid Construction

Synthetic oligodeoxynucleotides (GGAGA)_5_ and (TCTCC)_5_ were used to synthesize (GGAGA)_*n*_ · (TCTCC)_*n*_ by the use of the nontemplate PCR method [17]. The product was cloned into the *Eco*RV site of pBluescript SK(−) (Stratagene). Nucleotide sequencing showed that one clone contained GAG(AGGAG)_22_ in the *Kpn* I-to-*Sac* I direction and the clone was designated pSK-(AGGAG)_22_. A fragment containing the repeat was cut out with *Eco*R I and *Hin*d III and cloned in between the *Eco*R I and the *Hin*d III sites of pHC624 [18] to make pHC624-(AGGAG)_22_. Both plasmids were purified from *Escherichia coli* DH5*α* by Cesium-Ethidium banding [19].

### 2.2. Transcription

**Figure 1:**
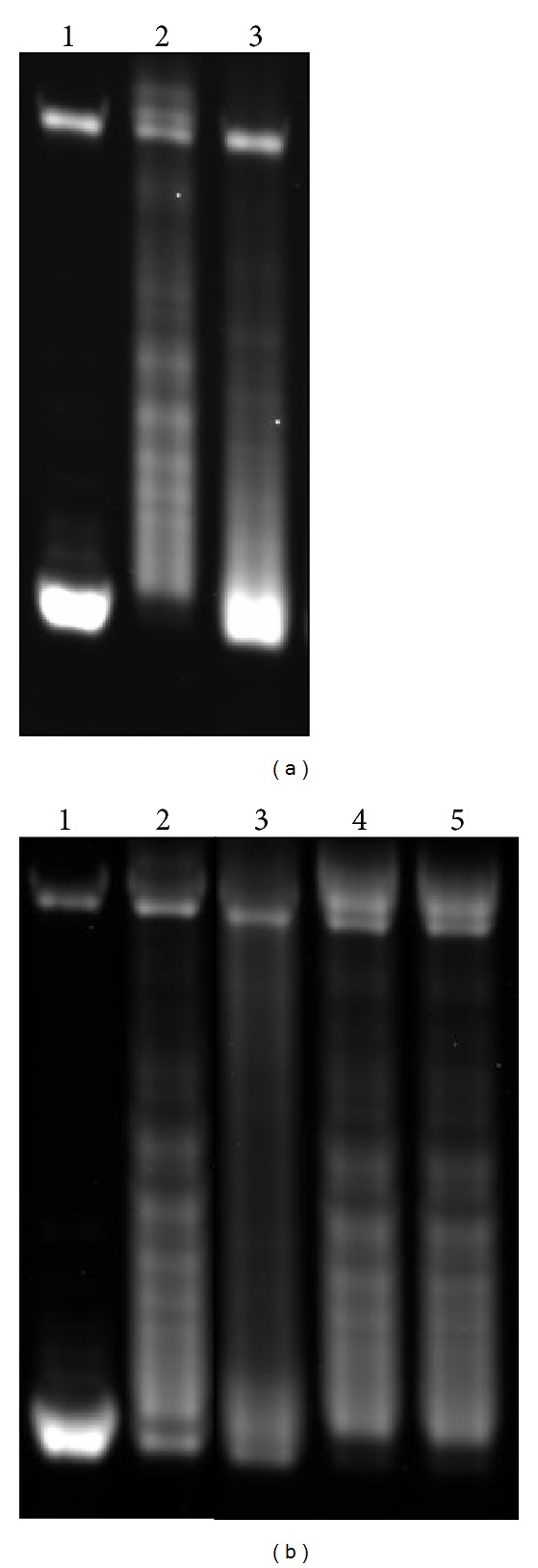
R-loop formation on pSK-(AGGAG)_22_ and effects of RNase T1 on the formation. A plasmid containing a (AGGAG)_22_ repeat was transcribed with T7 RNA polymerase and the effects on the topology of the plasmid were examined by agarose gel electrophoresis. (a) Lane 1: mock transcribed; lane 2: transcribed with T7 RNA polymerase; lane 3: transcribed with T3 RNA polymerase. (b) lane 1: mock transcribed, lane 2: transcribed with T7 RNA polymerase, Lane 3: transcribed with T7 RNA polymerase in the presence of 3 units of RNase T1; lane 4: transcribed with T7 RNA polymerase and ethanol-precipitated; and lane 5: transcribed with T7 RNA polymerase, ethanol-precipitated, and then incubated with 3 units of RNase T1.

Standard transcription reaction was carried out with 6 units of T7 RNA polymerase (Roche Diagnostics) on 0.3 *μ*g DNA (0.3 *μ*g for each DNA, when two DNA species were present) at 37°C for 20 min in 20 *μ*L of 50 mM NaCl, 40 mM Tris*·*HCl pH 8.0, 6 mM MgCl_2_, 2 mM spermidine, 10 mM DTT, and 0.6 mM each of ATP, CTP, GTP, and UTP [11, 12]. One *μ*L of 0.5 M EDTA pH 8.0 was added to terminate transcription and 2.5 *μ*L of 1 *μ*g/*μ*L RNase A (Sigma) was added [11, 12]. When the transcript was radiolabeled, [*α*-^32^P] ATP was also included. After incubation at 37°C for 1 h, the reaction was extracted with phenol/chloroform/isoamylalcohol. When RNase T1 was included (Figure 1(b)), transcription was carried out at an NaCl concentration of 150 mM for 20 min. One *μ*L of 0.5 M EDTA pH 8.0 was added and the reactions were incubated at 37°C for 5 min and extracted with phenol/chloroform/isoamylalcohol. Samples were electrophoresed in 1% agarose in 50 mM Tris*·*borate and 1 mM EDTA, DNA was stained with ethidium bromide, and the gel was photographed on a UV transilluminator or scanned on a Typhoon analyzer (GE Healthcare Life Science). For detection of the radiolabel, the agarose gel was dried on Hybond-N^+^ membrane (GE Healthcare Life Science), the radioactivity was recorded on an Image Plate (Fuji Photo Film), and the Plate was scanned on a Typhoon analyzer.

### 2.3. Chemical Probing of DNA Structure

Supercoiled pSK-(AGGAG)_22_ was relaxed with vaccinia topoisomerase I [20]. Five *μ*g of supercoiled or relaxed DNA was subjected to chemical modification in 200 *μ*L of 50 mM sodium cacodylate pH 8.0 and 6 mM MgCl_2_. Oxidation with potassium permanganate [21] was carried out at 0.9 mM for 2 min at 37°C and terminated by adding 10 *μ*L of *β*-mercaptoethanol. Modification with diethylpyrocarbonate [22] was started by adding 3 *μ*L of the reagent and vortexing, the reaction was incubated at 37°C for 10 min with one more vortexing during incubation, and 10 *μ*L of *β*-mercaptoethanol was added to terminate the modification. The DNAs were purified by the use of glass milk (Q-Biogene). Permanganate-oxidized DNA was digested with *Xho* I and dephosphorylated with calf intestine phosphatase. The reaction was extracted with phenol/chloroform/isoamylalcohol, and the DNA purified with glass milk. The DNA was end-labeled with [*γ*-^32^P] ATP and T4 polynucleotide kinase. After inactivating the kinase by heating at 65°C for 20 min, the DNA was digested with *Xho* I in the presence of 1 mg/mL spermidine [22]. After extraction with phenol/chloroform/isoamylalcohol, the samples were spun in S-400 HR columns (GE Healthcare Life Science) to remove unincorporated radiolabel and the small *Xho* I-*Kpn* I fragment. Diethylpyrocarbonate-treated DNA was similarly processed with *Not* I and *Sac* I. Recovered DNA was ethanol precipitated with 4 *μ*g of carrier DNA and air dried. The DNA was dissolved in 100 *μ*L of 1 M piperidine and 2 mM EDTA, heated at 90°C for 30 min, and vacuum-dried. The dried pellet was dissolved in 20 *μ*L of water and vacuum-dried. This process was repeated once, and the DNA was dissolved in formamide loading solution [19]. Maxam-Gilbert sequencing reactions were carried out by the standard procedures [19]. The samples were electrophoresed in 5% Long Ranger (Lonza)-urea sequencing gel. After washing-off of urea and drying of the gel, the radioactivity was recorded on an image plate and the plate was scanned on a Typhoon analyzer.

## 3. Results

### 3.1. Formation of an R-Loop at (AGGAG)_22_

In order to create a simplified model of S_*α*_, an AGGAG repeat was synthesized by the nontemplate PCR method [17] and cloned into the *Eco*R V site of pBluescript SK(−).One clone contained GAG(AGGAG)_22_ at the site in the direction that transcription from the T7 promoter would produce an AGGAG-repeat transcript. The plasmid, designated pSK-(AGGAG)_22_, purified from *E. coli* and negatively supercoiled, was transcribed with T7 RNA polymerase. Agarose gel electrophoresis showed extensive relaxation of the DNA (Figure 1(a), lane 2) indicative of R-loop formation. The relaxation was orientation-dependent (compare with transcription from T3 promoter; lane 3), as reported previously [11, 12]. In addition, when the repeat was moved into pBluescript KS(+) to reverse the orientation, transcription from the T3 promoter but not from the T7 promoter resulted in relaxation of the DNA (data not shown). These results showed that the AGGAG repeat was sufficient for R-loop formation, although the AGGAG repeat in the S_*α*_ region is preceded by a tantalizing GAGCT repeat (GenBank/EMBL/DDBJ entry D11468).

### 3.2. Inhibition of R-Loop Formation by RNase T1

In order to test whether the transcript was separated from and then reassociated with the template or the RNA stayed on the template, we included a ribonuclease (RNase) in the transcription mixture. If the RNA stays on the template, the RNase should not affect the R-loop formation. Although it was reported that inclusion of RNase A did not affect the formation [11, 12], RNase A is known to be specific to pyrimidines and thus would not be expected to digest the AGGAG-repeat RNA. Therefore, RNase T1, which is specific to purines, was chosen. When RNase T1 was present, the relaxation was diminished (Figure 1(b), lane 3). RNase T1 is known to cleave RNA in R-loops at lower salt concentrations. However, the R-loop was stable under the conditions used, as no change was observed when pre-formed R-loop was incubated with RNase T1 (lanes 4 and 5). Thus, the result indicated that the transcript was separated from and reassociated with the template and that the “separator” function of T7 RNA polymerase was not compromised on the CTCCT-repeat template, in agreement with results reported for other class-switch regions and a telomeric repeat [23].

### 3.3. R-Loop Formation in Trans and Its Dependence on DNA Supercoiling

**Figure 2:**
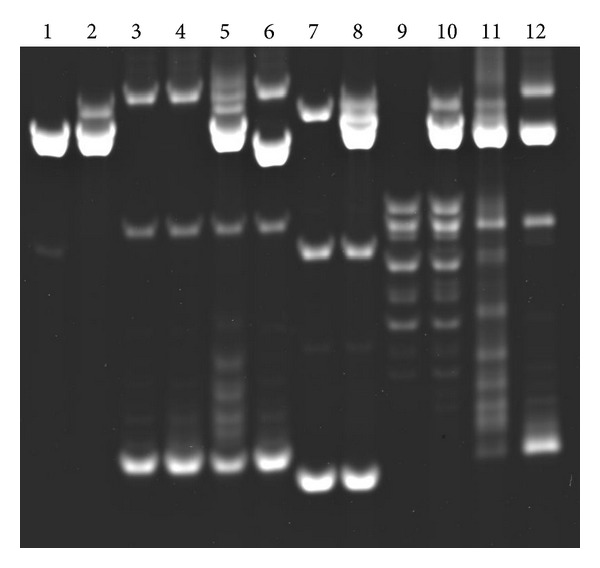
R-loop formation *in trans* and its dependence on supercoiling of the target DNA. RNA containing an AGGAG repeat was produced from linearized pSK-(AGGAG)_22_, and the effects on a T7 promoterless plasmid containing an AGGAG repeat, pHC624-(AGGAG)_22_, were examined by agarose gel electrophoresis. Lane 1: pSK-(AGGAG)_22_ linearized with *Xba* I; lane 2: linearized pSK-(AGGAG)_22_ transcribed with T7 RNA polymerase; lane 3: supercoiled pHC624-(AGGAG)_22_, which lacked T7 promoter; lane 4: supercoiled pHC624-(AGGAG)_22_ incubated in transcription mixture with T7 RNA polymerase; lane 5: supercoiled pHC624-(AGGAG)_22_ and linearized pSK-(AGGAG)_22_ transcribed with T7 RNA polymerase; lane 6: supercoiled pHC624-(AGGAG)_22_ and pBluescript SK(−) linearized with *Xba* I, transcribed with T7 RNA polymerase; lane 7: supercoiled pHC624; lane 8: supercoiled pHC624 and linearized pSK-(AGGAG)_22_ incubated in transcription mixture with T7 RNA polymerase; lane 9: pHC624-(AGGAG)_22_ relaxed with vaccinia topoisomerase I; lane 10: relaxed pHC624-(AGAAG)_22_ and linearized pSK-(AGGAG)_22_ transcribed with T7 RNA polymerase; lane 11: same as lane 5 and ethanol-precipitated; lane 12: same as lane 11 and treated with 90 units of *E. coli* RNase H (TaKaRa) in 40 mM Tris*·*HCl pH 7.7, 4 mM MgCl_2_, 1 mM DTT, 4% glycerol, and 0.003% bovine serum albumin at 37°C for 1 h.

The above result indicating reassociation suggested that the R-loop might form *in trans*. This possibility was explored by including in the transcription mixture a plasmid that contained the AGGAG repeat but no T7 promoter. The plasmid, pHC624-(AGGAG)_22_, was constructed by inserting the AGGAG repeat in a small cloning vector, pHC624 [18]. An AGGAG-repeat transcript was produced by transcribing linearized pSK-(AGGAG)_22_ with T7 RNA polymerase, and effects on the target pHC624-(AGGAG)_22_ were examined. The result is shown in Figure 2. Change in DNA topology was observed for supercoiled pHC624-(AGGAG)_22_ (Figure 2, lanes 4 and 5). The change was dependent on the presence of the AGGAG repeat both in the transcribed and the target plasmids (lanes 6–8). The change was reversed by treatment with RNase H (lanes 11 and 12), indicating that the change was due to the formation of an R-loop. No change in topology was observed for relaxed pHC624-(AGGAG)_22_ (lanes 9 and 10), indicating that the R-loop formation *in trans* depended on the superhelicity of the plasmid DNA.

**Figure 3:**
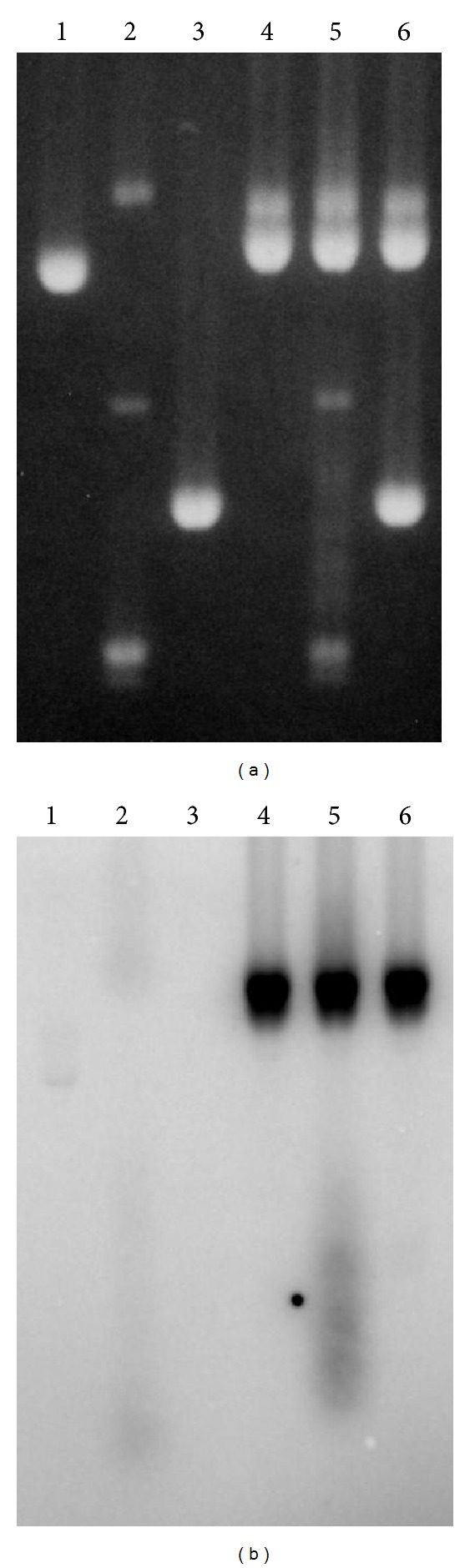
R-loop formation *in trans* detected by radiolabeling of the transcript. (a) Agarose gel stained with ethidium bromide and (b) autoradiogram of the same gel. Lane 1: pBluescript SK(−) linearized with *Xba* I; lane 2: supercoiled pHC624-(AGGAG)_22_; lane 3: pHC624-(AGGAG)_22_ linearized with *Sca* I; lane 4: pSK-(AGGAG)_22_ linearized with *Xba* I; lane 5: linearized pSK-(AGGAG)_22_ and supercoiled pHC624-(AGGAG)_22_; and lane 6: linearized pSK-(AGGAG)_22_ and linearized pHC624-(AGGAG)_22_. All DNAs were incubated in the transcription mixture with T7 RNA polymerase.

This R-loop formation *in trans* was further examined by linearizing pHC624-(AGGAG)_22_ and radiolabeling the transcript. Transcription of linearized pSK-(AGGAG)_22_ generated an extra band with a slightly reduced mobility (Figure 2). Radioactivity was found to be associated with this extra band (Figure 3, lanes 4–6), indicating the mobility shift was due to the presence of the R-loop. Transcription of linearized pSK-(AGGAG)_22_ resulted in relaxation of supercoield pHC624-(AGGAG)_22_ and radioactivity was associated with the relaxed species (lane 5). By contrast, little radioactivity was associated with pHC624-(AGGAG)_22_ when the DNA was linearized (lane 6). These confirmed the dependence of the R-loop formation *in trans* on supercoiling.

### 3.4. Formation of an Alternative Structure at the AGGAG Repeat by DNA Supercoiling

**Figure 4:**
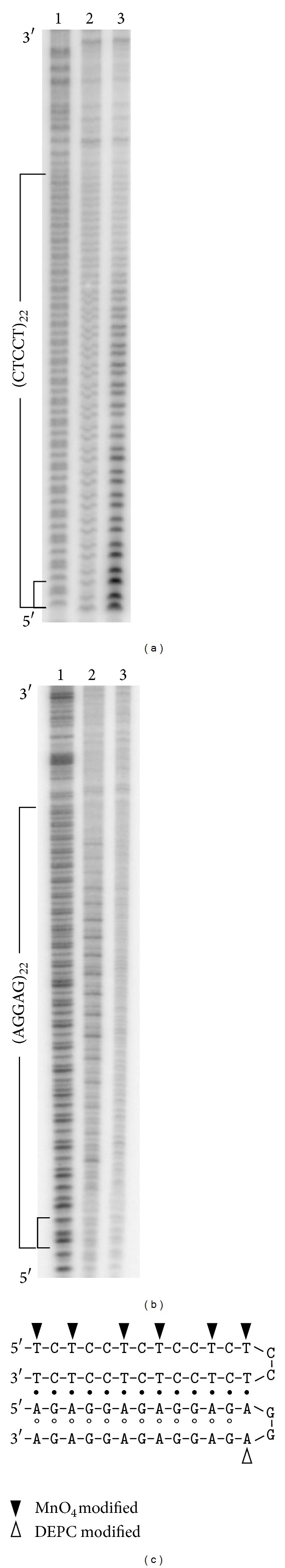
Supercoiling-induced structural change in the AGGAG repeat detected by chemical modification. Reactions of the Maxam-Gilbert type were carried out on relaxed and supercoiled pSK-(AGGAG)_22_ and analyzed by sequencing gel. (a) Oxidation of T residues by permanganate. Lane 1: Maxam-Gilbert C reaction; lane 2: relaxed DNA; lane 3: supercoiled DNA. The large bracket indicates the repeat and the small one, the 5′-most CTCCT unit. (b) Modification of A residues by diethylpyrocarbonate. Lane 1: Maxam-Gilbert G reaction; lane 2: supercoiled DNA; lane 3: relaxed DNA. The large bracket indicates the repeat and the small one, the 5′-most AGGAG unit. (c) An H-r3 structure consistent with the modification results. Filled circles indicate Watson-Crick base pairing, and open circles indicate triplex formation. Triangles indicate the bases expected to be reactive to the chemicals.

The results obtained thus far suggested that negative supercoiling induced a structural change and the CTCCT-repeat strand became accessible to the AGGAG-repeat transcript in the altered structure. Chemical modification was employed to detect the structural change.  Figure 4(a) shows the result for oxidation of T residues in the CTCCT-repeat strand by permanganate [21]. T residues were found to be oxidized more readily when the DNA was supercoiled than when relaxed. Permanganate is known to oxidize T residues (and C residues to a lesser extent) preferentially in single-stranded forms [21]. Therefore, the result indicated that supercoiling induced, a transition to an alternative structure in which the CTCCT-repeat strand was single stranded. The AGGAG-repeat strand was probed with diethylpyrocarbonate. The chemical is known to readily react with A (and less so with G) preferentially in single-stranded forms [22]. As shown in Figure 4(b), A residues in the central AGGAG units, especially the second A residues in the units, were more reactive to diethylpyrocarbonate when the DNA was supercoiled.

## 4. Discussion

One possible mechanism that allows reassociation would be zippering of RNA and DNA by backtracking of RNA polymerase [24]. However, in the current model of the elongation complex, backtracking dissociates the 3′ end of the transcript from the template [25]. This will limit the size of the R-loop to that of the transcription bubble, which is only several base pairs [2, 25]. A result on R-loop formation in *Saccharomyces cerevisiae* cells seems to suggest against backtracking as well, if the mechanism is assumed to be the same. Placing of a ribozyme sequence downstream of a *PHO5* was shown to reduce R-loop formation at the *PHO5* [10]. The result indicated that at least some population of the R-loop formed after the RNA polymerase had transcribed the ribozyme region, which was 52 bp. This would require the backtracking to start further downstream and to continue through the 52 bp region until reaching the *PHO5* to start forming an R-loop, both of which seem very unlikely. The present result of R-loop formation *in trans* also indicates that backtracking is not necessary, although the result does not exclude backtracking.

The present work showed that an R-loop at the AGGAG repeat formed *in trans* and the formation *in trans* was facilitated by negative supercoiling of the target DNA. The results of chemical probing of the repeat DNA under supercoiling were consistent with a structure in which the 5′ side in the CTCCT repeat was single-stranded (Figure 4). Thus, the R-loop formation *in trans* observed here is most likely to be the association of the AGGAG-repeat transcript with the CTCCT-repeat template that was made accessible by the structural conversion. Formation of alternative structures had previously been studied for (AGGAG)_6_GA [26]. The previous result of diethylpyrocarbonate modification at low pH was consistent with formation of an intramolecular triple helix. The present result of diethylpyrocarbonate modification was different from the previous one and, therefore, the alternative structure observed here is likely to be different from the previous one, which was a canonical H-form. Another structure had also been observed at neutral pH [26]. The regions of enhanced chemical modification were different between the previous and the present results, and thus, the structures are likely to be different. The difference is likely to be due to the number of the repeats and/or the presence of Mg(II), which was absent in the previous study, as dependence of the formation of alternative forms on the size of the homopurine*·*homopyrimidine stretch, metal ions, and pH has been well documented [27–29]. One structure consistent with our chemical modification results is depicted in Figure 4(c), which is an H-r3 isoform of the *H-form [27, 28]. Alternative fold-back points would result in stronger modification sites in the middle of the AGGAG strand.

Another possible mechanism consistent with the present findings would be one that involves opening of the duplex in the wake of the advancing RNA polymerase and ensuing hybridization of the transcript RNA to the template DNA, which has been proposed by Grabczyk et al. [30]. They have posited that negative supercoiling behind the advancing RNA polymerase induces triplex formation at GAA repeats, however, with no data to show the formation. The present study demonstrated that extensive helix opening does occur at an AGGAG repeat under negative supercoiling, indicating the repeat's potential of an alternative conformation. There is also circumstantial evidence linking R-loop formation at *E. coli rrnB* to supercoiling both *in vitro* and *in vivo* [31, 32]. It is debatable, however, whether the transient supercoiling behind the polymerase is strong enough to induce the conformation transition on small purified DNA molecules *in vitro*, especially because R-loop has been found to form on small linearized DNA that cannot be topologically constrained [12, 33]. Duquette et al. [23]studied G-loop formation in immunoglobulin class-switch regions and a telomeric repeat and have proposed that invasion of RNA into the duplex DNA initiates the formation. This general idea of RNA invasion into duplex is supported by the finding that a nick in the nontemplate strand can serve as an initiation site for R-loop at immunoglobulin S_*γ*3_ repeats *in vitro*: fraying of the nontemplate strand at the nick should increase the accessibility of the template strand to the transcript [33]. Finally, we would like to raise the possibility that the transient duplex separation by the RNA polymerase could be more persistent for some templates, which may involve interaction between DNA single strands and the RNA polymerase, and also the possibility that the nontemplate strand may contribute to the stability of the R-loop by the base interactions involved in triple helix formation [27, 28, 34, 35].

## 5. Conclusions

*In vitro* experiments showed that R-loop formed *in trans* at an AGGAG repeat when the DNA comprising the repeat was negatively supercoiled. Chemical modifications indicated that the duplex at the repeat opened under negative supercoiling. These results were the first to connect structural conversion, duplex opening, and R-loop formation for the same sequence, supporting the importance of the potential of duplex opening in R-loop formation.
